# Altered Expression of Type-1 and Type-2 Cannabinoid Receptors in Celiac Disease

**DOI:** 10.1371/journal.pone.0062078

**Published:** 2013-04-19

**Authors:** Natalia Battista, Antonio Di Sabatino, Monia Di Tommaso, Paolo Biancheri, Cinzia Rapino, Paolo Giuffrida, Cinzia Papadia, Chiara Montana, Alessandra Pasini, Alessandro Vanoli, Francesco Lanzarotto, Vincenzo Villanacci, Gino R. Corazza, Mauro Maccarrone

**Affiliations:** 1 Department of Biomedical Sciences, University of Teramo, Teramo, Italy; 2 European Center for Brain Research (CERC)/Santa Lucia Foundation, Rome, Italy; 3 Department of Internal Medicine, Fondazione IRCCS Policlinico S. Matteo, Centro per lo Studio e la Cura della Malattia Celiaca, University of Pavia, Pavia, Italy; 4 Gastroenterology Unit, Parma University Hospital, Parma, Italy; 5 Department of Molecular Medicine, Fondazione IRCCS Policlinico S. Matteo, Centro per lo Studio e la Cura della Malattia Celiaca, University of Pavia, Pavia, Italy; 6 Department of Gastroenterology, Spedali Civili di Brescia, Brescia, Italy; 7 Department of Pathology, Spedali Civili di Brescia, Brescia, Italy; 8 Center of Integrated Research, Campus Bio-Medico University of Rome, Rome, Italy; University of Cincinnati, United States of America

## Abstract

Anandamide (AEA) is the prominent member of the endocannabinoid family and its biological action is mediated through the binding to both type-1 (CB_1_) and type-2 (CB_2_) cannabinoid receptors (CBR). The presence of AEA and CBR in the gastrointestinal tract highlighted their pathophysiological role in several gut diseases, including celiac disease. Here, we aimed to investigate the expression of CBR at transcriptional and translational levels in the duodenal mucosa of untreated celiac patients, celiac patients on a gluten-free diet for at least 12 months and control subjects. Also biopsies from treated celiac patients cultured *ex vivo* with peptic-tryptic digest of gliadin were investigated. Our data show higher levels of both CB_1_ and CB_2_ receptors during active disease and normal CBR levels in treated celiac patients. In conclusion, we demonstrate an up-regulation of CB_1_ and CB_2_ mRNA and protein expression, that points to the therapeutic potential of targeting CBR in patients with celiac disease.

## Introduction

Cannabinoid receptors (CBR) belong to the large superfamily of heptahelical Gi/o protein coupled receptors [1]. Type-1 (CB_1_) and type-2 (CB_2_) cannabinoid receptors act as main molecular targets of anandamide (AEA) and mediate the biological action of this lipid by activating distinct signalling pathways [2]. CB_1_ has been mainly found in cells and tissues of central nervous system [3], whereas CB_2_ is localized preferably on peripheral and immune cells [4], but it has been identified also in neuronal cells [5], [6]. Experimental studies demonstrated the presence of CBR in various sections of the gastrointestinal tract and a dysregulation of their expression has been reported in several gut pathologies, including diarrhoea [7], colon cancer [8], inflammatory and irritable bowel diseases [9], [10]. High AEA levels in the duodenal mucosa of untreated celiac disease (UCD) patients in comparison to treated celiac disease (TCD) patients and control subjects (CS) [11] are likely due to an altered *N*-acylphosphatidyl-ethanolamine specific phospholipase D (NAPE-PLD) activity [12], and might self-induce an increase of CBR, as a fine mechanism of regulation common to many diseases [11], [13], [14]. Indeed, immunofluorescence analyses showed that CB_1_ protein is strongly expressed in duodenum biopsies from UCD patients [11], whereas CB_2_ is up-regulated, both at transcriptional and translational levels, in small bowel biopsies obtained from children with celiac disease [15]. Here, we investigated CBR mRNA and protein as well as functional activity levels in the duodenal mucosa of UCD and TCD patients, and CS. Moreover, we explored the effect of the peptic-tryptic digest of gliadin (PT-gliadin) on CBR expression in organ culture biopsies taken from TCD patients.

## Materials and Methods

5 - (1,10 - Dimethylheptyl) - 2 - [(*1R,5R*) - hydroxy - (*2R*) - (3-hydroxypropyl) - cyclohexyl]phenol (CP55940) was purchased from Sigma Chemicals (St. Louis, MO, USA) and [^3^H]CP55940 (174.6 Ci/mmol) was from PerkinElmer Life Sciences (Boston, MA, USA).

### Ethics statement

This study was approved by the Ethical Committee of Spedali Civili of Brescia. Informed written consent to participate in this study was given by patients.

### Patients and tissues

Biopsy samples were collected from the second part of the duodenum of 16 patients affected by uncomplicated UCD (9 males and 7 females, mean age 40.6 years, range 19–71). The diagnosis was based on positivity of serum antiendomysial antibodies associated with the typical histopathological lesions, namely villous atrophy, increased intraepithelial lymphocyte infiltration and crypt hyperplasia. Six patients showed a Marsh IIIc lesion and 10 showed a Marsh IIIb lesion. Duodenal biopsies were also collected from 17 patients affected by uncomplicated celiac disease on a gluten-free diet for at least 12 months (7 males and 10 females, mean age 32.4 years, range 18–81), all negative for serum antiendomysial antibodies and with a substantially normal duodenal mucosal architecture. Finally, duodenal biopsies were also collected from 19 CS (8 males and 11 females, mean age 46.7 years, range 18–69) undergoing endoscopy for functional dyspepsia, negative for antiendomysial antibodies and with normal histology. Some of the biopsies were processed for routine histology or were embedded in OCT Tissue-Tek (Sakura Finetek, Torrance, CA, USA) snap frozen and then stored at −70°C; others were used for organ culture or were homogenized for immunoblotting analysis.

### Organ culture

Biopsy specimens from TCD patients, placed on grids in the central well of an organ culture dish, were cultured in an airtight container with 95% O_2_/5% CO_2_ at 37°C in the absence or presence of 1 mg/ml PT-gliadin (Frazer III fraction, Sigma-Aldrich) in RPMI-1640 medium (Gibco, Invitrogen, Paisley, UK) supplemented with 10% HL-1 (Lonza BioWhittaker, Verviers, Belgium), 100 U/ml penicillin and 100 mg/ml streptomycin [16]. After 24 h culture, biopsies were snap frozen and stored at −70°C.

### Quantitative real-time reverse transcriptase-polymerase chain reaction (qRT-PCR) analysis

Total RNA was extracted from biopsies using the RNeasy extraction kit (Qiagen, Crawley, UK), as suggested by the manufacturer. QuantiTect Reverse Trascription kit (Qiagen, Crawley, UK) was used to produce cDNA from 1 µg of purified RNA. 40 ng of the first strand of cDNA was used for amplification (in triplicate) in 15 µl reaction solution, containing 7.5 µl QuantiFast SYBR Green PCR (Qiagen) and 10 pmol of each primer (Table 1). The following qRT-PCR program was used: 95°C for 5 min PCR initial activation step; 40 amplification cycles at 95 °C for 10 s, 60°C for 30 s. The target transcripts were amplified by means Opticon™ 2 continuous fluorescence detection system (MJ Research, San Francisco, CA, USA). β-Actin was used as housekeeping gene for quantity normalization.

**Table 1:** Oligonucleotide sequences of CB_1_, CB_2_ and β-actin used for detection by qRT-PCR analysis.

mRNA target	Forward	Reverse
CB_1_	5′- CCTTTTGCTGCCTAAATCCAC-3′	5′-CCACTGCTCAAACATCTGAC-3′
CB_2_	5′-TCAACCCTGTCATCTATGCTC-3′	5′-AGTCAGTCCCAACACTCATC-3′
β-actin	5′-TGACCCAGATCATGTTTGAG-3′	5′-TTAATGTCACGCACGATTTCC-3′

### Western blotting

Western blotting was performed according to standard procedures [17]. In brief, tissue samples were lysed in ice-cold lysis buffer (10 mM EDTA, 50 mM pH 7.4 Tris-HCl, 150 mM sodium chloride, 1% Triton-X-100, 2 mM phenylmethylsulfonyl fluoride, 2 mM sodium orthovanadate, 10 mg/ml leupeptin and 2 mg/ml aprotinin) and the amount of protein was determined by the Bio-Rad Protein assay (Bio-Rad Laboratories, Hemel Hempstead, UK). Equivalent amounts of protein were loaded in each lane and run on 10% sodium dodecyl sulphate-polyacrylamide gel electrophoresis under reducing conditions. Proteins were transferred to nitrocellulose membranes (Bio-Rad Laboratories, Hercules, California, USA), that were blocked with 10% non-fat dried milk and 5% bovine serum albumin for 2 h, and then incubated overnight at 4°C with the rabbit polyclonal antibodies specific for CB_1_ (1∶200 dilution) or CB_2_ (1∶1000 dilution) receptors (both from Abcam, Cambridge, UK). Membranes were rinsed and incubated with the appropriate horseradish peroxidase-conjugated secondary anti-rabbit antibody (diluted 1∶4000; Dako, High Wycombe, UK) in blocking solution. Detection was performed using ECL Plus detection reagents (Amersham, Little Chalfont, UK). Blots were stripped and analyzed for β-actin, as an internal loading control, using a rabbit anti-human β-actin antibody (Abcam). Protein expression levels were quantified by densitometric analysis, using the ImageJ software after quantity normalization with β-actin.

### Confocal microscopy

Immunofluorescence staining of 4 µm thick cryostat sections of OCT-embedded biopsy specimens fixed in cold acetone was performed using the same anti-CB_1_ or CB_2_ antibodies used in Western blotting (1∶100 dilution), followed by a FITC-conjugated secondary goat anti-rabbit antibody (1∶500 dilution; Abcam). Nuclei were counterstained by DAPI (1∶1000 dilution; Sigma-Aldrich, Poole, UK). Appropriate isotype control antibody was included on parallel sections. The sections were mounted with coverslips using Glycergel Mounting Medium (Dako), and were analyzed using a laser scanning confocal microscope (FluoView FV1000; Olympus, Center Valley, PA, USA). Images (1,024×1,024 pixels) were acquired using an oil immersion lens (60×1.4 NA Plan-Apochromat; Olympus).

### Enzyme-linked immunosorbent assay (ELISA)

Biopsy homogenates (20 µg/well) were coated overnight and were reacted with rabbit anti-CB_1_ or anti-CB_2_ polyclonal antibodies (1∶250 dilution) (both from Cayman Chemicals, Ann Arbor, MI, USA). Goat anti-rabbit antibody conjugated to horseradish peroxidase (1∶5000 dilution, Santa Cruz Biotechnology, Santa Cruz, CA, USA) was used as second antibody, and color development of the horseradish peroxidase reaction was followed at 405 nm using 1-Step Ultra TMB ELISA substrate (Pierce, Rockford, IL, USA).

### Receptor binding assay

CBR functional activity was evaluated by using the MultiScreenHTS 96-well Plates for binding assays [18]. Briefly, biopsies were homogenated in 50 mM Tris–HCl (pH 7.4) and 20 µg of lisate were incubated with [^3^H]CP55.940 (2.5 nM). Incubations were performed in 0.2 ml reaction buffer (50 mM Tris-HCl, 2 mM Tris-EDTA, 3 mM MgCl_2_, 5 mg/ml BSA, pH 7.4). The filters were washed and transferred to vials, containing 0.1% Triton X-100 (0.5 ml) in 3.5 ml liquid scintillation cocktail (Ultima Gold XR, Perkin Elmer Life Sciences, Boston, MA, USA). Unspecific binding was determined in the presence of cold agonists (1 µM CP55940), and all binding data were expressed as fmol per mg of protein.

### Statistical analysis

Results were expressed as mean±SEM of at least triplicate experiments. Data were analyzed by means of Prism 5 program (GraphPad Software, San Diego, CA, USA) using the one-way analysis of variance (ANOVA) followed by Bonferroni's *post hoc* analysis. A level of p<0.05 was considered statistically significant.

## Results

### *In situ* mucosal CB_1_ and CB_2_ expression by immunofluorescence

**Figure 1:**
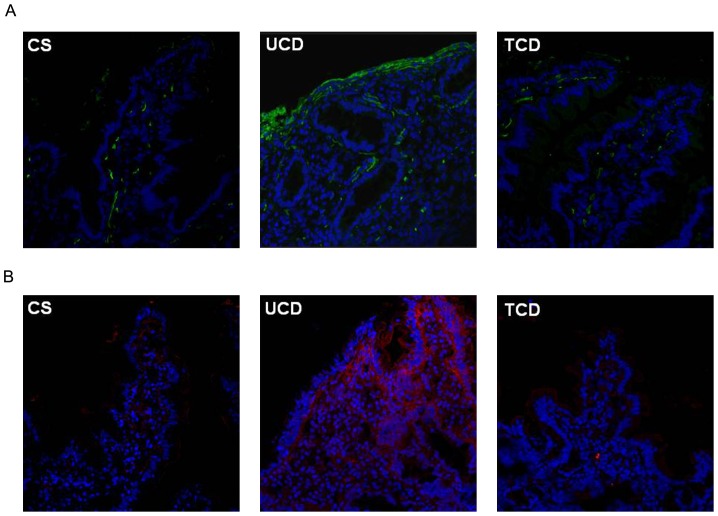
CB_1_ and CB_2_ immunofluorescence by confocal miscroscopy. Expression of CB_1_ (A) and CB_2_ (B) in the duodenal mucosa of a patient with untreated celiac disease (UCD), a patient with treated celiac disease (TCD) and a control subject (CS). Numerous CB_1_- and CB_2_-positive cells were evident both in the epithelium and lamina propria of UCD patients, while only few mononuclear cells were scattered in the lamina propria of TCD patients and CS. Data are representative of staining performed in 10 UCD patients, 10 TCD patients and 10 CS. Original magnification ×40.

We first determined the expression of CB_1_ (Figure 1A) and CB_2_ (Figure 1B) by confocal immunofluorescence in the duodenal mucosa of UCD and TCD patients, and CS. Numerous CB_1_- and CB_2_-positive cells were evident both in the epithelium and lamina propria of UCD patients, while in TCD patients and CS positivity for CB_1_ and CB_2_ was limited to a few mononuclear cells scattered in the lamina propria.

### Mucosal CB_1_ and CB_2_ mRNA and protein levels

**Figure 2:**
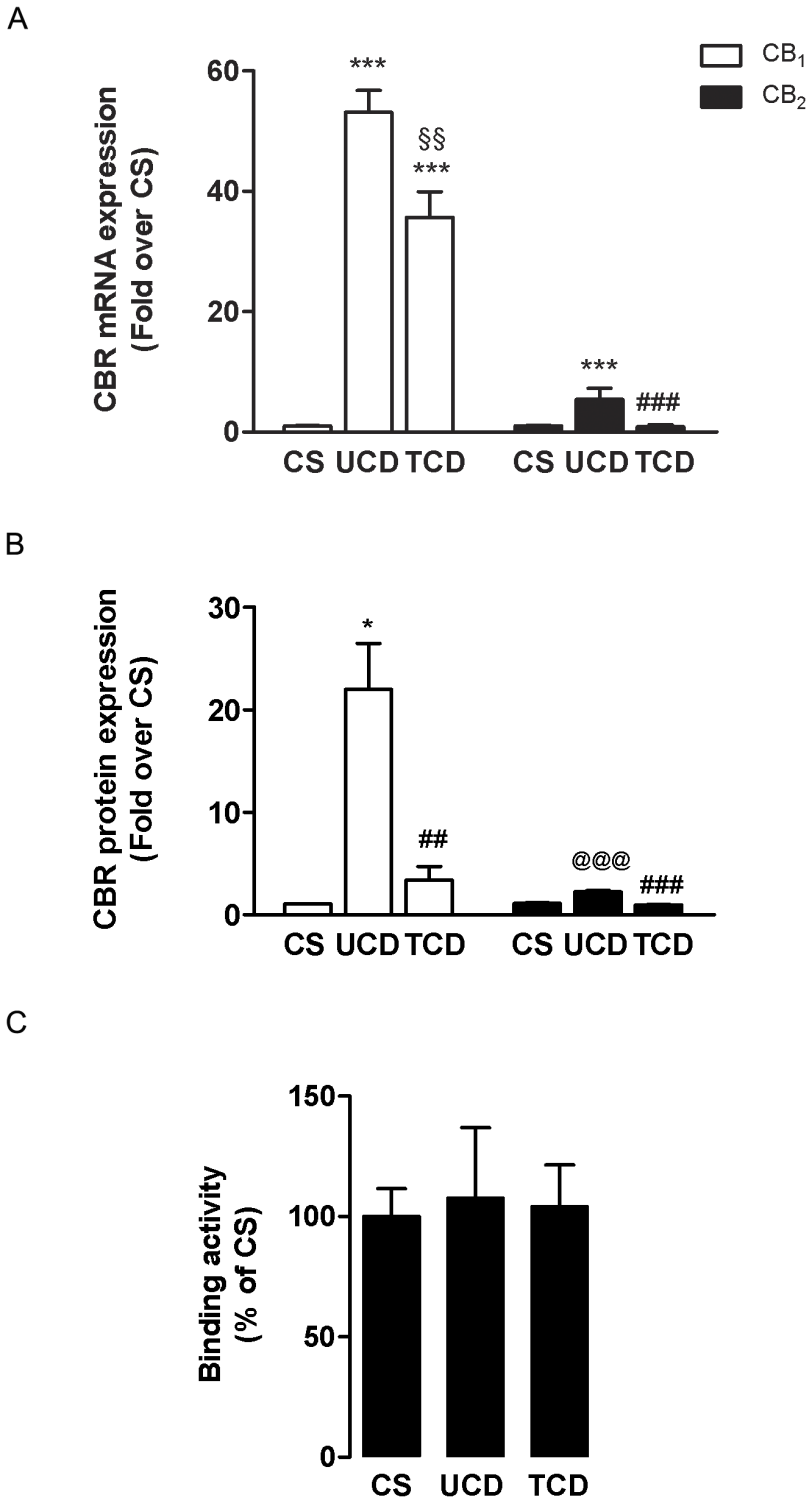
CB_1_ and CB_2_ gene and protein expression levels. A) qRT-PCR analysis of CB_1_ and CB_2_ in the duodenal mucosa of untreated celiac disease (UCD) patients, treated celiac disease (TCD) patients and control subject (CS) (n = 6). ***p<0.0001 *vs* CS, ^§§^p<0.001 *vs* UCD, for CB_1_; ^###^p<0.0001*vs* UCD, for CB_2_. B) CB_1_ and CB_2_ levels measured by ELISA in the biopsies of untreated celiac disease (UCD) patients, treated celiac disease (TCD) patients and control subjects (CS) (n = 6). *p<0.05 *vs* CS, ^##^p<0.01 *vs* UCD, for CB_1_@@@p<0.0001 *vs* CS, ^###^p<0.0001 *vs* UCD, for CB_2_. C) CB_1_ and CB_2_ binding activity in the duodenal mucosa of untreated celiac disease (UCD) patients, treated celiac disease (TCD) patients and control subjects (CS) (n = 4).

CB_1_ and CB_2_ mRNA and protein expression was investigated through qRT-PCR (Figure 2A) and ELISA (Figure 2B), respectively. These analyses demonstrated the presence of both CBR in biopsies collected from the duodenum of UCD, TCD and CS. Remarkably, CB_1_ mRNA levels increased significantly in the mucosa of UCD and TCD patients compared to CS (p<0.0001), although CB_1_ decreased in patients after remission following a gluten-free diet (p<0.001). Additionally, the expression of CB_2_ mRNA was almost 10-fold higher in UCD patients than in healthy mucosa (p<0.0001), and was lower than that of TCD patients (p<0.0001). In keeping with the qRT-PCR results, ELISA analysis revealed an higher expression of CB_1_ protein in UCD patients with respect to CS (p<0.05), and lower CB_1_ protein levels in TCD patients compared with UCD subjects (p<0.01). Incidentally, no statistically significant difference was found between TCD and CS groups. CB_2_ protein expression showed a similar trend compared to CB_1_ protein, showing an increased level in UCD patients compared with CS (p<0.0001) and a significant reduction in TCD patients (p<0.0001), where CB_2_ protein levels were comparable to those of CS.

### CBR binding assay

Binding assays were performed with the synthetic agonist [^3^H]CP55.940, that has high affinity for both CB_1_ and CB_2_ receptors [4]. The results reported in Figure 2C show that biopsies obtained from the three patients' groups were able to bind the radioligand to similar extents: 186±21 fmol per mg of protein (CS), 200±54 fmol per mg of protein (UCD) and 193±32 per mg of protein (TCD).

### *Ex vivo* mucosal CB_1_ and CB_2_ expression

**Figure 3:**
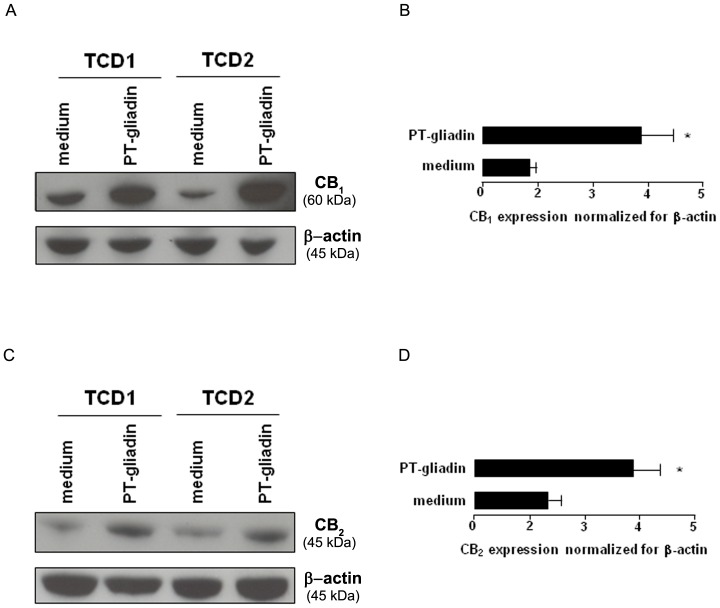
*Ex vivo* effect of PT-gliadin on mucosal expression of CB_1_ and CB_2_. CB_1_ (A) and CB_2_ (B) protein levels were detected by immunoblotting in treated celiac disease (TCD) biopsies incubated for 24 h with or without 1 mg/ml peptic tryptic digest of gliadin (PT-gliadin). Each example is representative of experiments performed in 11 TCD patients. Panels C and D show densitometry of CB_1_ (C) and CB_2_ (D) expression normalised for β-actin. *p<0.001 *vs* medium. Results are mean±SD.

To investigate the effect of PT-gliadin on mucosal CB_1_ and CB_2_ expression, we measured by immunoblotting CB_1_ and CB_2_ protein levels in mucosal biopsies from TCD patients cultured *ex vivo* in the absence or presence of PT-gliadin. Mucosal biopsies cultured with PT-gliadin showed significantly (p<0.001) higher levels of CB_1_ (up to ∼4-fold) in comparison to biopsies treated with medium only (Figure 3A, B). Similarly, mucosal biopsies cultured with PT-gliadin showed significantly (p<0.001) higher levels of CB_2_ in comparison to biopsies treated with medium only (Figure 3C, D).

## Discussion

In this study, we demonstrated that CB_1_ and CB_2_ expression is up-regulated, both at transcriptional and translational level, in active celiac mucosa. In addition, we reported that the *ex vivo* incubation of treated celiac biopsies with PT-gliadin significantly increased the expression of mRNA and protein of both receptors. These *in vivo* and *ex vivo* data are in agreement with previous studies, showing an alteration of the endocannabinoid system in the duodenal mucosa of UCD patients [11], [12]. Moreover, we have recently reported that mRNA, protein and activity levels of the main enzyme responsible for AEA synthesis, NAPE-PLD, are increased in UCD mucosa compared to TCD and normal mucosa [12], a finding that could provide a possible explanation for the increased AEA concentration previously shown in the mucosa of UCD patients [11]. The presence of CB_1_ and CB_2_ receptors has been demonstrated in different segments of the gastrointestinal tract and their involvement in disorders where intestinal inflammation and gut dysfunctions take place has been confirmed in several *in vitro* human studies [19], [20]. In particular, it has been ascertained that CBR tone is relevant in controlling important intestinal functions [19], [21] and that a number of gastrointestinal diseases are related to genetic alterations of CBR [10], [15], [22]–[23]. Indeed, a polymorphism of CB_1_-encoding *Cnr1 gene* was found to modulate the susceptibility to Crohn's disease and ulcerative colitis [22], and was associated with patients affected by inflammatory bowel disease [10], [23]. More recently, in a cohort of children the Q63R variant of the CB_2_-encoding *Cnr2 gen*e was shown to increase more than 6-fold the risk of celiac disease [15]. Based on these studies and considering that alterations in CBR expression might be a specific response to a pathological condition, we investigated the presence of CB_1_ and CB_2_ in the duodenal mucosa of celiac patients through molecular, immunochemical and functional analyses. Our *in vivo* data showed that mRNA and protein levels of both CB_1_ and CB_2_ receptors are remarkably increased in UCD mucosa compared to TCD mucosa and normal mucosa. It is noteworthy that in TCD patients CB_2_, but not CB_1_, levels were reverted to normal values, pointing to CB_2_ rather than CB_1_ as main molecular target in celiac disease. Moreover, e*x vivo* experiments on organ culture confirmed that gluten-induced damage is responsible for this increase, at least at the protein level. Higher levels of CB_1_ in the duodenal mucosa of UCD patients have been already reported in a previous study, suggesting that their up-regulation could be an adaptative mechanism to counteract the inflammation [11]. Here, we also point to the role of CB_2_ in the control of gut inflammation, and this is in keeping with the notion that this receptor is mainly expressed on immune cells and is implicated both in infectious [24], [25] and inflammatory [26] diseases of the gastrointestinal tract. The relevance of CB_2_ in celiac disease has been highlighted in a recent investigation performed on biopsies from Italian children [15]. Although the results are in line with ours, the authors evidenced only a slight increase of CB_2_ mRNA, likely due to the age range of patients. Indeed, it is not surprising that during human development CBR expression is age-, as well as gender-, dependent [27]. Although it remains to be explained why CBR functionality is similar in all three groups, our results open perspectives to future investigations on epigenetic mechanisms, such as DNA methylation and histone modification, in the regulation of CBR expression in celiac disease. In this context, we should recall that our group has recently reported a correlation between selective *faah* gene expression alteration and DNA methylation in Alzheimer's disease patients [28], highlighting how epigenetic studies might be helpful in the identification of new therapeutic targets within the endocannabinoid system. Moreover, according to very recent papers [15], [29]–[31], it would be interesting to ascertain whether CBR gene-by-phenotype associations can be found also in this pathology, or CBR polymorphism in childhood might be considered a symptom of predisposition for celiac disease risk. In conclusion, our findings together with those published in a previous study [12], suggest that an abnormal modulation of the endocannabinoid system, both at CBR and AEA levels, may be implicated in the pathogenesis of celiac disease. Further studies are needed to ascertain whether targeting these changes might have a therapeutic role, at least in those patients who are no longer responsive to gluten-free diet.
